# Cardioprotective Effect of Acetylsalicylic Acid in the Myocardial Ischemia-Reperfusion Model on Oxidative Stress Markers Levels in Heart Muscle and Serum

**DOI:** 10.3390/antiox11081432

**Published:** 2022-07-23

**Authors:** Piotr Frydrychowski, Marcin Michałek, Iwona Bil-Lula, Elżbieta Chełmecka, Alina Kafel, Agnieszka Noszczyk-Nowak, Dominika Stygar

**Affiliations:** 1 Department of Internal Medicine and Clinic of Diseases of Horses, Dogs and Cats, Faculty of Veterinary Medicine, Wrocław University of Environmental and Life Sciences, Grunwaldzki Square 47, 50-366 Wrocław, Poland; marcin.michalek@upwr.edu.pl; 2 Department of Medical Laboratory Diagnostics, Division of Clinical Chemistry and Laboratory Hematology, Wrocław Medical University, 50-556 Wrocław, Poland; iwona.bil-lula@umw.edu.pl; 3 Department of Statistics, Department of Instrumental Analysis, Faculty of Pharmaceutical Sciences in Sosnowiec, Medical University of Silesia, 40-055 Katowice, Poland; echelmecka@sum.edu.pl; 4 Department of Natural Sciences, Institute of Biology, Biotechnology and Environmental Protection, University of Silesia in Katowice, 40-007 Katowice, Poland; alina.kafel@us.edu.pl; 5 Department of Physiology in Zabrze, Faculty of Medical Sciences in Zabrze, Medical University of Silesia in Katowice, 40-055 Katowice, Poland; dstygar@sum.edu.pl

**Keywords:** myocardial ischemia-reperfusion, acetylsalicylic acid, oxidative stress markers

## Abstract

Heart failure occurs in increased oxidative stress conditions, which contribute to the progression of pathological changes. Orally or intravenously administered acetylsalicylic acid (ASA, aspirin) is typically used in human patients with acute myocardial ischemia. The study used an experimental porcine ischemia-reperfusion model to evaluate the potential cardioprotective effect of intracoronary administered ASA on myocardial ischemia-reperfusion injury. The cardioprotective effect of ASA was evaluated by measuring selected oxidative stress markers levels in infarcted and non-infarcted myocardium 14 days after the procedure, and three times in serum, before the procedure, during the reperfusion process, and after 14-day recovery. The results showed that intracoronary administrated ASA reduced the oxidative stress. The level of oxidative stress, measured with the non-enzymatic markers total antioxidant capacity (TAC), total oxidative status (TOS), and malondialdehyde (MDA), and the enzymatic markers glutathione peroxidase (GPx), glutathione reductase (GR), and glutathione S-transferase (GST), in heart tissue was significantly higher in a control group injected with saline. The level of oxidative stress in serum, measured with TAC, TOS, oxidative stress index (OSI), and lipofuscin (LF), was also higher in the control group than in animals injected with ASA. The confirmed cardioprotective effect of intracoronary administered ASA provides the foundation for further studies on ASA intracoronary application, which may lead to the development of a new therapy for the treatment of ischemia-reperfusion complications in humans.

## 1. Introduction

Cardiovascular diseases (CVDs) and their complications are the most common causes of death in industrialized countries [1]. Over the past several decades, investigations into human heart failure and animal models of heart failure have provided substantial evidence that oxidative stress increases during heart failure and contributes to disease progression [2]. Oxidative stress (OS) is described as the disturbance in the equilibrium of pro- and antioxidants in favor of the prooxidants. Oxidative stress contributes significantly to the atherogenic processes [3,4]. In isolated heart cells, it changes the gene expression and induces cell death that accompanies myocardial remodeling and heart failure [2]. Hyperlipidemia, hypertension, heart diseases, and other conditions linked to CVD are associated with prooxidant overproduction or endogenous antioxidant deficiencies [5,6,7]. Prooxidants comprise free radical species or non-radical species mediating peroxidation and include, among others, reactive oxygen species (ROS) and reactive nitrogen species (RNS) [4]. In acute myocardial infarction (MI), reactive oxygen species (ROS) are generated in the ischemic myocardium, especially after reperfusion. Reactive oxygen species directly injure the cell membrane and cause cell death [8]. Myocardial infarction and heart failure are CVDs considered valid research targets for prooxidant–antioxidant imbalance [4]. The therapeutic effects of antioxidants on heart failure progression have already been reported [2].

Unfortunately, the mechanism of myocardial damage caused by ischemia and subsequent reperfusion (IR) is yet to be fully understood, but inflammation and platelet activation to post-reperfusion injury play a significant role [9,10]. Aspirin (acetylsalicylic acid, ASA) is an anti-inflammatory and antiplatelet drug administered orally to patients with the acute coronary syndrome. Its protective role results from inhibiting cyclooxygenases (COXs) that metabolize arachidonic acid and produce prostaglandin. Although ASA does not affect cardiovascular function directly, it seems to effectively protect against many cardiovascular pathologies such as atherosclerosis, ischemic heart disease, and myocardial infarction. Intracoronary ASA administration during acute ischemia was hoped to reduce the inflammatory response and, in consequence, block platelets [11]. Studies showed that intravenous ASA administration did not reduce the size of post-ischemia necrosis. However, it inhibited the conversion of PGG and PGH prostaglandins to thromboxane A2, and their further conversion to the cardioprotective PGE, PGD, and PGI2 prostaglandins, by blocking platelet cyclooxygenase [12]. Other studies showed that ASA protects the heart muscle against damage caused by energy metabolism disturbances occurring during an ischemic episode. The cardioprotective effect results from blocking the production of prostaglandins and stabilizing the cell membrane [11]. Other studies have showed that ASA inhibits prostacyclin production in heart tissue by blocking COX, which exacerbates myocardial dysfunction induced by IR episode [12]. Studies on intracoronary drug administration and the effectiveness of intravenous vs. intracoronary drug administration have showed that anticoagulants (GP IIb/IIIa receptor inhibitors) are much more effective when administered via an intracoronary route. They reduce thrombus growth and improve coronary blood flow [13,14]. Intracoronary administration of abciximab (an antiplatelet drug, the glycoprotein IIb/IIIa inhibitor) improved myocardial reperfusion, as reflected by a decrease in enzymatic markers in patients with myocardial infarction [15]. It can be assumed that intracoronary ASA administration may more effectively improve the coronary blood flow in infarction compared to oral or intravenous (peripheral) administration.

In the past decade, various animal models of human diseases have been developed for cardiovascular research on myocardial regeneration and therapy [16,17]. One of the most commonly used models is the porcine model of ischemia-reperfusion [18]. The swine (*Sus scrofa domestica*) heart is anatomically similar to the human heart. Lesions induced by ischemia-reperfusion in the porcine model represent well the injury occurring in patients who have suffered from an acute myocardial infarction. The standard treatment procedure in myocardial ischemia is the restoration of blood flow in the coronary vessels (PCI procedure) [19]. However, the risk of damage due to reperfusion after previous ischemia is considerable [20].

The present study investigated the potential cardioprotective effects of intracoronary administered aspirin (ASA) on the ischemia-reperfusion model using selected oxidative stress markers. To fulfil the goal, we studied levels of non-enzymatic and enzymatic oxidative stress markers in serum and heart muscle tissue collected from infarcted and non-infarcted parts of the left ventricle of the heart. Confirming the cardioprotective effect of aspirin administered to the coronary vascular system in the porcine model would provide the basis for proposing this new pharmacological strategy to clinicians. The results from our study would help to expand the prevention and treatment methods for heart damage after coronary arteries ischemia and reperfusion episodes in human patients.

## 2. Materials and Methods

### 2.1. Ethical Statement and Permissions, Animals

The research complied with the National Institute of Health guidelines for the care and use of laboratory animals. The experimental protocol was approved by the Local Ethics Committee for Animal Experiments at Wroclaw University of Environmental and Life Sciences, Poland (protocol no. 081/2019, approved on 11 December 2019).

Female pigs (*Sus scrofa domestica*) of the Polska Biała Zwisłoucha breed (*n* = 13, 16–20 weeks old, 33–44 kg) were purchased from the Experimental Station of the National Research Institute of Animal Production in Żerniki Wielkie (Poland). The use of female pigs stems from our previous studies in a porcine model. Female pigs exhibit greater resilience to stress associated with daily handling, grooming, and new housing conditions. In addition, they are more resistant to perioperative stress. Females are also less aggressive towards other animals and staff and quickly habituate to animal handling staff.

The animals were housed and maintained in the same controlled conditions, with a diet conforming to nutritional standards and water ad libitum. All animals were habituated to grooming activities before the study started.

### 2.2. Study Groups

Animals were divided into the following two groups: control group (*n* = 6) and ASA group (*n* = 7). Animals from both groups were subjected to the acute myocardial ischemia protocol. During the reperfusion period, after inducing myocardial ischemia, the animals from the control group received an intracoronary injection of NaCl, while animals from the ASA group received an intracoronary injection of acetylsalicylic acid. All animals subjected to the experiment survived the induction the myocardial ischemia, the reperfusion period, and the 14-day recovery time (100% survival rate).

#### Evaluation of the Animals’ Health Status

The pigs’ health status before the induction of myocardial ischemia, during the reperfusion period, and after the 14-day recovery time (before euthanasia) was evaluated based on clinical examination and biochemical (aspartate aminotransferase (AST), urea, creatinine, and fibrinogen levels) and morphological blood tests (white blood cells (WBC) and red blood cells (RBC) count).

### 2.3. Protocol for Inducing Acute Myocardial Ischemia in a Porcine Model

#### 2.3.1. Premedication

The animals fasted for 12 h before the procedures started. Premedication was performed with intramuscular injection of 10 mg/kg b.m. of ketamine (Vetaketam 100 mg/mL, Vet-Agro Sp. z.o.o., Lublin, Poland), 0.3 mg/kg b.m. of midazolam (Midanium 5 mg/mL, Polfa Warszawa S.A., Warsaw, Poland), and 0.03 mg/kg b.m. of medetomidine (Sedator 1 mg/mL, Eurovet Animal Health BV, Bladel, The Netherlands).

#### 2.3.2. General Anesthesia Induction and Maintenance

After immobilization, the animals were placed in a sternal lying posture, and the marginal vein of the ear was catheterized in order to obtain vascular access. General anesthesia was induced with an intravenous bolus (2 mg/kg b.m.) of propofol (Provive 10 mg/mL, Claris Lifesciences UK Ltd., Crewe, Cheshire, UK). Then, the animals were intubated with a size 8 endotracheal tube (Tracheal Tube type Murphy, SUMI, Sulejówek, Poland) and mechanically ventilated for the remainder of the procedure using 100% oxygen and a closed gas system with a carbon dioxide absorber. The initial phase of ventilation was pressure-controlled, and after evaluating the ventilation parameters and capnometry, the ventilation switched to volume-controlled mode. The oxygen flow was maintained at 2 L/min, with a tidal volume of 10 mL/kg b.m. and respiratory rate of 12/min. Ventilator (Primus, Dräger Medical AG & Co. KGaA, Lübeck, Germany) settings were adjusted individually to obtain end-tidal CO_2_ concentration between 35 and 45 mmHg.

General anesthesia was maintained with isoflurane (Forane, Abbott Laboratories, Warsaw, Poland) inhaled via a calibrated vaporizer (Vapor 2000, Dräger Medical AG & Co. KGaA, Lübeck, Germany). The concentration of isoflurane (1.5–2.5%) in 100% oxygen was monitored (Lifepak 12, Medtronic, Redmond, WA, USA) and adjusted according to the capnometry value, to achieve 1 MAC (minimum alveolar concentration). Analgesia was achieved by injecting an intravenous bolus (10 µg/kg b.m.) of fentanyl (Fentanyl WZF 50 µg/mL, Polfa Warszawa S.A., Warsaw, Poland) and continued with its intravenous infusion at 10 µg/kg b.m./h rate.

#### 2.3.3. Hemodynamic Stability Maintenance

In the intra- and perioperative period, fluid therapy was performed using constant rate infusion (CRI) of a multi-electrolyte solution (Optilyte, Fresenius Kabi Polska Sp. z o.o., Kutno, Poland) at 6–12 mL/kg b.m./h rate, based on the animal’s hydration status and response to medication.

During the procedure, the following vital functions of the animals were monitored (Lifepak 12, Medtronic, Redmond, WA, USA): internal body temperature, saturation, pulse, and blood pressure. Additionally, the heart rhythm was constantly tracked using a 12-lead ECG (BTL-08 MT Plus ECG, BTL Industries Ltd., Stevenage, Herefordshire, UK).

Hypotension events occurring during the procedure were stabilized with 10 mL/kg b.m boluses of lactated Ringer’s solution (Solutio Ringeri Lactate Fresenius, Fresenius Kabi Polska Sp. z o.o., Kutno, Poland) and 3–5 mL/kg b.m. boluses of hydroxyethyl starch 130/0.4 (HES; Voluven, Fresenius Kabi Deutschland GmBH, Bad Homburg vor der Höhe, Germany). In the absence of adequate response of the circulatory system to fluid resuscitation, continuous intravenous infusion of dopamine (Dopaminum Hydrochloricum WZF 40 mg/mL, Polfa Warszawa S.A., Warsaw, Poland) was administered at an initial dose of 4 µg/kg b.m./min, and afterwards, the dosage was adjusted to the animal’s clinical status and vital parameters. Additionally, the Trendelenburg position was periodically employed to rebalance the circulatory system function.

#### 2.3.4. Myocardial Ischemia Induction

Acute myocardial ischemia was obtained following previously described protocols [21,22,23] with minor modifications.

Percutaneous vascular access, via the vascular sheath (6F diameter, Balton, Warsaw, Poland), to the femoral artery was gained using a puncture needle (21G, Balton, Warsaw, Poland) and ultrasound guidance (F37, Hitachi Aloka Medical Ltd., Mure, Mitaka-shi, Tokyo, Japan). After inserting the guide catheter (Launcher, JL 3.5 curvatures, 6F diameter, Medtronic, Santa Rosa, CA, USA), a 6000 UI heparin bolus (Heparinum WZF 5000 UI/mL, Polfa Warszawa S.A., Warsaw, Poland) was injected, and the coronary arteries were assessed by angiography.

Afterwards, an angioplasty guidewire (BMW, 3 m, 0.014″, Abbott, Santa Clara, CA, USA) was introduced through the catheter and positioned in the proximal segment of the left anterior descending artery (LAD) under the control of fluoroscopy (Symbol, General Medical Merate SpA, Seriate, Italy). A 3.0 × 10 mm angioplasty balloon (Sprinter, OTW model, Medtronic, Santa Rosa, CA, USA) was located on the guidewire and inflated to a pressure of 6 atm for 30 min to obtain complete LAD closure. The occlusion of the LAD was verified by angiography, and myocardial infarction was recognized by ST-segment elevation on the 12-lead ECG.

#### 2.3.5. Reperfusion and Acetylsalicylic Acid (ASA) Treatment

Reperfusion was achieved by deflating the angioplasty balloon and restoring blood supply to the ischemic part of the myocardium. Ten minutes before emptying the balloon, animals belonging to the ASA group were injected intracoronary with 150 mg of acetylsalicylic acid (Kardegic 0.5 g 500 mg/5 mL, Sanofi-Aventis s.r.o., Praha, Czech Republic), whereas animals in the control group were injected intracoronary with the same volume of 0.9% NaCl. The acetylsalicylic acid dose was determined based on recommendations regarding its use in patients with acute coronary syndrome [24]. The invasive procedure of myocardial infarction induction and reperfusion was concluded 15 min after deflating the angioplasty balloon.

### 2.4. Postoperative Treatment and the Recovery Time

Postoperatively, an adequate analgesic treatment was provided by intramuscular injections of buprenorphine (Bupaq Multidose 0.3 mg/mL, Richter Pharma AG, Wels, Austria) (0.01 mg/kg b.m., every 8 h) for 3 days, metamizole (Pyralgivet 500 mg/mL, Vet-Agro Sp. Z.o.o., Lublin, Poland) (50 mg/kg b.m., every 24 h) for 3 days, and meloxicam (Metacam 5 mg/mL Boehringer Ingelheim Vetmedica GmBH, Ingelheim/Rhein, Germany) (0.4 mg/kg b.m., every 24 h) for 2 days. Afterwards, until the 14th day after the procedure, the animals were housed and maintained as before the experiment.

### 2.5. Blood Sample Collection

Blood for biochemical and morphological analyses was collected in plastic tubes with a clot activator or EDTA, respectively. The first sample was collected shortly after cannulating the right femoral artery before inducing ischemia. The second sample was collected during the reperfusion period, 15 min after deflating the angioplasty balloon and 25 min after ASA administration. The third sample was collected before the animal was euthanized. Blood for morphological analyses was collected before inducing ischemia and before euthanizing the animals.

### 2.6. Euthanasia and Heart Tissue Collection

The animals were euthanized 14 days after acute myocardial ischemia induction. The animals were premedicated according to the protocol described above (intramuscular administration of ketamine, midazolam, and medetomidine). Then, propofol was administered through the marginal vein of the ear. After inducing anesthesia, the third blood sample for biochemical analyses was collected through the right femoral artery. Euthanasia was performed by an intravenous administration of a 100 mg/kg b.m.of pentobarbital and sodium pentobarbital mixture (Morbital 133.3 mg/mL + 26.7 mg/mL, Biowet Puławy Sp z o.o., Puławy, Poland).

After completing the euthanasia procedure, each animal from the control and from the ASA group was subjected to an autopsy, during which two samples (100 mg each) of the left ventricular myocardium were collected, one from the infarcted (the necrotic area induced by the ischemia) and one from the non-infarcted area (visible healthy tissue) of the left ventricular myocardium. The cardiac tissue was collected to 1 mL of a homogenizing buffer with protease inhibitors. Then, it was homogenized (1:10 *w/v*) in 0.9% NaCl with a glass homogenizer (Potter-Elvehjem PTFE, Sigma-Aldrich, Darmstadt, Germany) and sonicated (Virsonic 100, VirTis, Gardiner, NY, USA). The lysate was centrifuged for 10 min, at 4000× *g* rpm, at 4 °C, and treated as one independent sample. The tissue samples were then frozen and stored at −80 °C until the analysis.

### 2.7. Oxidative Stress Marker Analysis

Oxidative stress markers were analyzed in the left ventricle samples (infarcted and non-infarcted) and in the blood serum samples. In the left ventricle samples, the following non-enzymatic oxidative stress markers were analyzed: total antioxidant capacity (TAC), total oxidative status (TOS), oxidative stress index (OSI), and malondialdehyde (MDA) concentration, and activity of the enzymatic oxidative stress markers glutathione peroxidase (GPx), glutathione reductase (GR), and glutathione S-transferase (GST). Concentrations of the non-enzymatic oxidative stress markers TAC, TOS, OSI, and lipofuscin (LF) were analyzed in the serum samples.

#### 2.7.1. Total Antioxidant Capacity

Total antioxidant capacity (TAC) was assessed using a Randox TAS assay kit (Randox Co., Crumlin, County Antrim, UK). ABTS (2,2′ azino-di-(3-ethylbenzothiazoline sulphonate) was incubated with a peroxidase (metmyoglobin), hydrogen peroxide, and the sample to obtain the radical cation (ABTS+), in which a blue-green color can be measured at 600 nm. Suppression of the color was compared to the standard for TAC measurement assays (Trolox), and the results were expressed as a Trolox equivalent (mmol/L) [25].

#### 2.7.2. Total Oxidative Status

Total oxidative status (TOS) was determined as described by Erel [25]. The method uses the oxidation of Fe^2+^ to Fe^3+^ in an acidic medium. The produced Fe^3+^ ions react with xylene orange and form a colorful blue-purple complex, detected at 560 nm. The TOS level was determined against the calibration curve using H_2_O_2_ as the standard, and the results were expressed in μmol/L.

#### 2.7.3. Oxidative Stress Index

Oxidative stress index (OSI) was expressed as the total oxidant status to total antioxidant capacity (TOS/TAC) ratio and expressed in arbitrary units [26].

#### 2.7.4. Malondialdehyde Concentration

Malondialdehyde (MDA) concentration in samples was determined using the method of Ohkawa et al. with thiobarbituric acid. MDA concentration was detected spectrophotometrically (at 515 nm for excitation and at 552 nm for emission), calculated from the standard curve prepared using 1,1,3,3-tetraethoxypropane and expressed in μmol/L [27].

#### 2.7.5. Glutathione Peroxidase Activity (EC 1.11.1.9)

Glutathione peroxidase (GPx) activity was assessed using the kinetic method described by Mannervik. The decrease in NADPH concentration was monitored spectrophotometrically at 340 nm for 10 min, and GPx activity was expressed in IU/mg protein [28].

#### 2.7.6. Glutathione Reductase Activity (EC 1.8.1.7)

Glutathione reductase (GR) activity was assessed using the kinetic method described by Carlberg and Mannervik. The decrease in NADPH concentration in the samples was monitored at 340 nm for 10 min, and GR activity was expressed in IU/mg protein [29].

#### 2.7.7. Glutathione S-Transferase Activity (EC 2.5.1.18)

Glutathione S-transferase (GST) activity was evaluated using the kinetic method described by Habig and Jakoby [30] with 1-chloro-2,3-dinitro-benzene as a reaction substrate. GST activity was expressed in IU/mg protein.

#### 2.7.8. Lipofuscin Concentration

Lipofuscin (LF) concentration was determined as described by Tsuchida et al. [31]. The serum sample was mixed with ethanol-ether (3:1, *v/v*), shaken, and centrifuged. The fluorescence intensity was determined at 345 nm (absorbance) and 430 nm (emission). LF concentration was expressed in relative lipid extract fluorescence (RF), where 100 RF corresponds to the fluorescence of 1 μg/mL quinidine sulfate in 0.1 N sulfuric acid.

### 2.8. Statistical Analysis

Statistical analysis was performed using Statistica, version 13 (TIBCO Software Inc., Palo Alto, CA, USA). Statistical significance was set at *p* < 0.05. Data distribution was assessed using the Shapiro–Wilk test. The mean ± standard deviation (SD) was calculated for normally distributed data. The median with upper and lower quartile (Me (Q_1_; Q_3_)) was determined for data with a skewed distribution. Data with a skewed distribution were log-transformed before further analyses. A two-way ANOVA analysis with contrast analysis was used for concentrations comparison in the heart tissues samples (oxidative stress markers) and in the blood (health status markers). An analysis of variance for repeated measures was used to compare concentrations in the serum samples (oxidative stress markers) and in the blood (health status markers), and Mauchly’s test was used to assess the sphericity assumption.

## 3. Results

### 3.1. Biochemical and Morphological Blood Tests

The results of biochemical and morphological blood tests, except for fibrinogen, were within the normal ranges at each phase of the experiment (Table 1).

We did not observe statistically significant differences in AST (*p* = 0.470), WBC (*p* = 0.861) and RBC (*p* = 0.870) concentrations between all stages of the experiment. Additionally, we did not observe statistically significant differences between control and ASA groups for these three parameters (*p* = 0.801 for WBC, and *p* = 0.964 for RBC).

We observed statistically significant differences in urea concentrations at different experiment stages (*p* < 0.05), with the highest urea concentration during the reperfusion. We found no statistically significant differences between the control and ASA group (*p* = 0.369), and no interaction between these two variables (*p* = 0.968).

We noted that creatinine levels differed significantly between the experiment stages (*p* < 0.01), but found no differences between the control and ASA group (*p* = 0.562) nor any interactions between variables (*p* = 0.977). Creatinine levels in the control group differed between before ischemia and before euthanasia (*p* < 0.05) and during reperfusion and before euthanasia (*p* < 0.05). In the ASA group, the creatinine levels differed only between the reperfusion and euthanasia stages (*p* < 0.05).

Fibrinogen levels were significantly different at each stage of the experiment (*p* < 0.001), but no differences were detected between groups of animals (*p* = 0.509). For individual animal groups, differences were found for fibrinogen levels before ischemia and during reperfusion (p_control_ < 0.001, p_ASA_ < 0.001) and for reperfusion and euthanasia (p_control_ < 0.01, p_ASA_ < 0.05) stages. Fibrinogen levels in the control group before euthanasia were minimally above the reference range, while at other stages of the experiment, they were within the normal range.

### 3.2. Heart Muscle Tissue

**Figure 1:**
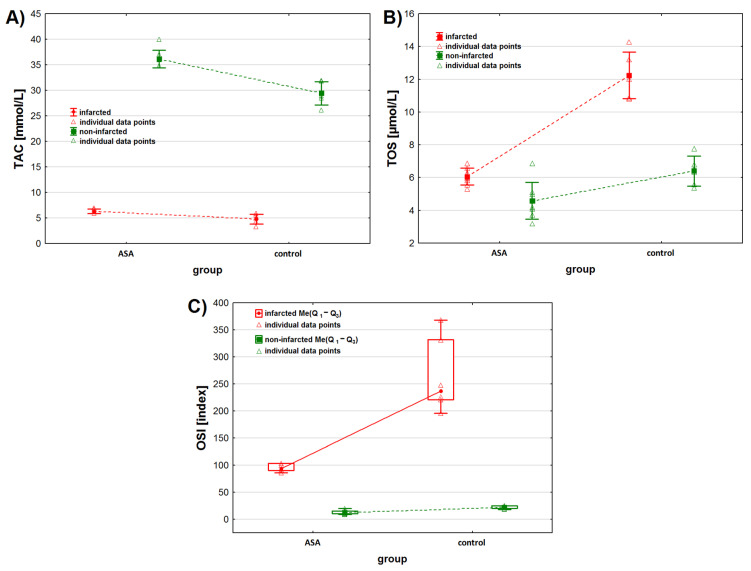
Oxidative stress markers levels in infarcted and the non-infarcted tissues collected from the left ventricle of the heart of pigs (*n* = 13) subjected to a myocardial ischemia-reperfusion protocol assessing the effectiveness of intracoronary administered acetylsalicylic acid (ASA): (**A**) total antioxidant capacity (TAC) [mmol/L], (**B**) total oxidative status (TOS) [µmol/L] and (**C**) oxidative stress index (OSI) [index]. Results in (**A**,**B**) are presented as mean ± 95% confidence interval and in (**C**) as median (lower, upper quartile) and minimum and maximum. In all figures, triangular markers represent the raw data. For the reader’s convenience, the points are connected with dashed lines.

We observed statistically significant differences in TAC levels between the infarcted and non-infarcted tissue of the left ventricle (*p* < 0.001). In addition, we found that TAC levels were significantly higher in the heart tissue collected from animals treated with intracoronary-administered ASA (Table 2 and Figure 1A). Moreover, the TAC level depended on the interaction between the infarction presence and drug administration. The contrast analysis showed that, regardless of the status of the collected heart tissue—infarcted or non-infarcted—TAC levels were statistically different after the administration of drugs (ASA or NaCl) (Table 3). No statistically significant differences in TAC levels were observed between the infarcted tissue of animals from the ASA and control groups. Contrast analysis showed that the TAC level in the non-infarcted tissue was higher than in the infarcted tissue (Table 3). Moreover, when considering the infarcted heart tissue only, we found differences in TAC levels between animals treated with ASA and animals from the control group (*p* < 0.001).

We found statistically significant differences between TOS levels in the infarcted and the non-infarcted tissue of the left ventricle, regardless of the treatment received (*p* < 0.001). Higher TOS levels were observed in the infarcted heart tissue (Table 2, Figure 1B). Also, we found statistical differences in TOS levels between the ASA group and the control group (*p* < 0.001) when not taking the status of the collected tissues (infarcted vs. non-infarcted) into consideration. TOS levels depended on the interaction between the two analyzed parameters. Contrast analysis showed that myocardial infarction increased TOS levels. TOS levels in the infarcted tissue were higher than in the non-infarcted tissue and depended on the treatment the animals received (*p* < 0.001 for the control group and *p* < 0.05 for the ASA group). When analyzing the myocardial tissue status individually, we found that, for the non-infarcted tissue, TOS levels were higher in the control group, while for the infarcted tissue they were higher in the ASA group.

We found statistically significant differences in OSI values between the infarcted and non-infarcted tissue, regardless of ASA or saline use (*p* < 0.001). Higher OSI values were noted for the infarcted heart tissue (Table 2, Figure 1C). We found statistically significant differences in OSI values between the ASA and control group, regardless of the status of the collected heart (*p* < 0.001). The highest OSI values were observed in the control group. OSI values also depended on the interaction between the analyzed factors (*p* < 0.05). Contrast analysis showed that myocardial infarction significantly increased OSI values. OSI values in the infarcted tissue were higher than in non-infarcted tissue and depended on the treatment the animals received (*p* < 0.001, respectively). We found higher OSI values in the non-infarcted tissue of the control group.

**Figure 2:**
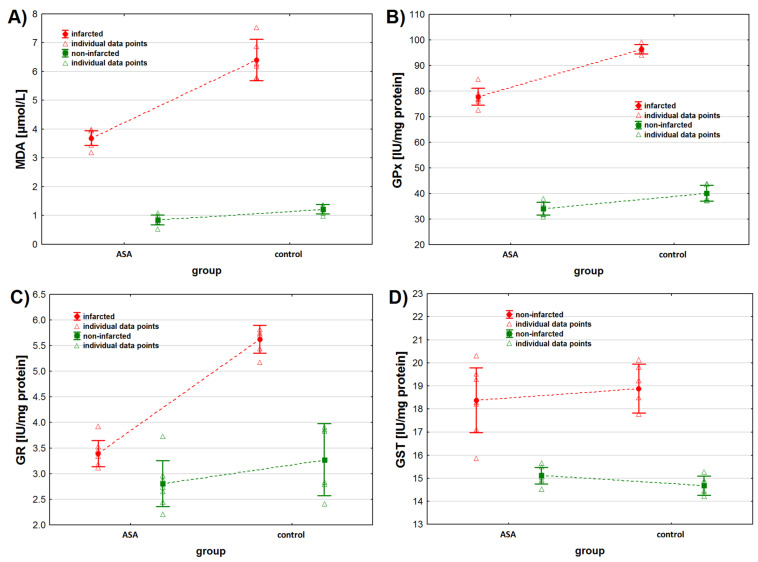
Oxidative stress markers levels in infarcted and the non-infarcted tissues collected from the left ventricle of the heart of pigs (*n* = 13) subjected to a myocardial ischemia-reperfusion protocol assessing the effectiveness of intracoronary administered acetylsalicylic acid (ASA): (**A**) malondialdehyde (MDA) concentration [µmol/L], (**B**) glutathione peroxidase (GPx) activity [IU/mg protein], (**C**) glutathione reductase (GR) activity [IU/mg protein] and (**D**) glutathione S-transferase (GST) activity [IU/mg protein]. The results are presented as mean ± 95% confidence interval and triangular markers represent the raw data. For the reader’s convenience, the points are connected with dashed lines.

As for MDA, we found higher MDA concentrations in the non-infarcted tissue when considering the status of the tissue only (*p* < 0.001) (Table 2, Figure 2A). Similarly, when considering the animals’ treatment only, we found higher MDA concentrations in the tissues collected from animals from the control group (*p* < 0.001). However, the interaction between both tested factors was also found to be statistically significant. Contrast analysis showed that MDA concentration was higher in the infarcted than in the non-infarcted heart tissue of animals from both study groups (*p* < 0.001 for both groups). The analysis of the non-infarcted tissue showed no difference in MDA concentration between animals injected with ASA and NaCl (*p* = 0.092), but higher MDA concentrations were observed in the infarcted heart tissue sampled from control group (*p* < 0.001) (Table 2 and Table 3).

As for GPx activity, we found higher GPx activity in the infarcted tissue compared to the non-infarcted tissue and in the heart tissue collected from the control animals compared to the ASA treated animals (Table 2, Figure 2B) when considering these two factors separately (tissue status and treatment type). However, the interaction between the tissue status and the treatment type was also found to be significant (*p* < 0.001). Contrast analysis showed the same pattern for data distribution when considering both factors together. GPx activity was higher in the infarcted tissue and in animals from the control group (Table 2 and Table 3).

We noted higher GR activity in the infarcted tissue than in the non-infarcted tissue (*p* < 0.001), when considering the tissue status only (Table 2, Figure 2C). No statistical differences in GR activity were noted between the ASA and the control group, when considering the type of treatment only (Table 2 and Table 3, *p* = 0.075), but an interaction between these two factors was noted (*p* < 0.001). When considering the treatment individually (ASA vs. control), we noted higher GR activity in the infarcted tissue. We noted higher GR activity in the heart tissue collected from the control group of animals, when considering the tissue status individually (infarcted vs. non-infarcted) (Table 2 and Table 3).

GST activity was higher in the infarcted tissues both in ASA and in the control group (Table 2 and Table 3, Figure 2D). We found no differences in GST activity, individually in the infarcted and in the non-infarcted tissue, when comparing the results in the ASA and in the control group.

### 3.3. Serum

**Figure 3:**
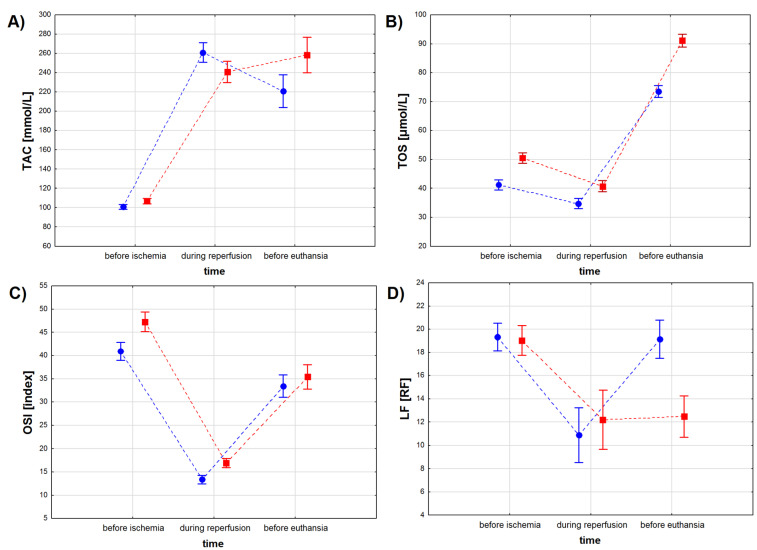
Oxidative stress marker levels in the serum of pigs subjected to a myocardial ischemia-reperfusion protocol assessing the effectiveness of intracoronary administered acetylsalicylic acid (ASA): (**A**) total antioxidant capacity (TAC) [mmol/L], (**B**) total oxidative status (TOS) [µmol/L], (**C**) oxidative stress index (OSI) [index] and (**D**) lipofuscin (LF) concentration [RF]. The results are presented as mean ± 95% confidence interval and, for the reader’s convenience, the points are connected with dashed lines.

TAC levels depended on the sampling time (*p* < 0.001), when not considering the treatment type, but no differences in TAC levels were found between the ASA and the control group (*p* = 0.079), when considering the treatment type only (Table 4, Figure 3A). However, we found a significant interaction between the treatment type and the treatment time (*p* < 0.001). The lowest TAC levels were observed for both study groups before the infarction, with lower TAC values for the ASA than for the control group except during reperfusion (*p* < 0.01). TAC values increased in both study groups during reperfusion, with the highest values observed for the ASA group (*p* < 0.001). During the recovery period, TAC values decreased in the ASA group, while no change in TAC values was noted in the control group (*p* = 0.073). Before euthanasia, TAC values in the control group were higher than in the ASA group (*p* < 0.001) (Table 4).

TOS values depended on the treatment type and the treatment time, when considering both factors individually and when considering both factors together (Table 4 and Table 5, Figure 3B). Higher TOS values were noted for serum sampled from animals from the control group compared to results obtained from the ASA group. Myocardial infarction decreased TOS values in both groups, while the recovery period (sampling before euthanasia) increased TOS values to higher levels than before infarction.

OSI values depended both on treatment type and treatment time (*p* < 0.001 for both factors), but not on the interaction between these two factors (*p* = 0.072) (Table 4, Figure 3C). OSI values decreased during the reperfusion period and increased during recovery, but to lower values than those noted before ischemia. Before myocardial infarction and during the reperfusion period, OSI values were higher in the control group, while during recovery no statistically significant differences in OSI values were found between the animals injected with ASA and the animals injected with NaCl (*p* = 0.251) (Table 5).

LF concentration depended both on treatment type and treatment time (*p* < 0.001 for both factors) and on the interaction between these two factors (Table 4, Figure 3D). We found no differences in LF concentration between the study groups (*p* = 0.766) before the infarction protocol started (Table 4 and Table 5). Higher LF values were noted in the control group (*p* < 0.01) during the reperfusion period and in the ASA group during recovery (*p* < 0.001). We found no differences in LF concentration measured for the reperfusion and for the recovery period in samples collected from the animals from the control group (*p* = 0.811).

## 4. Discussion

Oxidative stress activates many cellular responses characteristic of heart failure. These changes include not only cellular hypertrophy, changes in gene expression, and cell death [32,33], but also alterations in the turnover and properties of the extracellular matrix [34]. Classic stimuli for ventricular remodeling like wall stress, inflammatory cytokines, and neurohormones (catecholamines and angiotensin II) appear to induce cellular changes partially via oxidative or nitrosative stress [2,35,36]. The pathways activating the various cellular phenotypes of hypertrophy and apoptosis involve one or more stress-responsive protein kinases, many of which are activated by reactive oxygen species (ROS) [33,37]. The present work studied the cardioprotective effect of intracoronary-administered acetylsalicylic acid (ASA) in the ischemia-reperfusion model. The effect was measured with non-enzymatic and enzymatic oxidative stress marker level in the animals’ serum and the heart muscle tissue collected from infarcted and non-infarcted parts of the left ventricle. Here, we report that (i) non-enzymatic (TAC, TOS, MDA) and enzymatic (GPx, GR, GST) oxidative stress markers in heart tissue were significantly higher in the control group compared to the ASA group; (ii) intracoronary administered ASA reduced oxidative stress levels compared to the control group injected with NaCl; (iii) ASA showed a protective effect and reduced OS in the infarcted heart tissue when compared to the non-infarcted myocytes of the left ventricle; (iv) TAC, TOS, OSI, GPx, and GR levels were significantly related to the health status of the heart tissue (healthy or infarcted tissue), and type of drug used (ASA or NaCl in the control group); (v) infarction significantly increased OS in heart tissue compared to healthy heart tissue in the ASA group (except for TOS and GR markers) and in the control group; (vi) despite the type of drug used (ASA or NaCl), infarction decreased or increased TAC, MDA, and GST levels in the heart tissue in the same way. The results obtained for serum showed higher levels of oxidative stress in the control group than in the ASA group and that (vii) time is a significant factor influencing levels of all oxidative stress markers (TAC, TOS, OSI, and LF) measured in animals’ serum.

The health status of the animals was evaluated by clinical examination and blood tests at each stage of the experiment. Almost all assayed biochemical and morphological parameters ranged within the reference norms at all experiment stages. Only fibrinogen levels were minimally elevated in control pigs before euthanasia, indicating that the inflammatory process had started. However, we found no differences between the control and ASA groups for health status parameters at any stage of the experiment. Only healthy animals were eligible for the study, and MI induction and ASA treatment did not significantly affect the health status of the pigs.

Despite remarkable improvements in strategies for myocardial infarction and subsequent heart failure treatment, the understanding of heart failure pathogenesis remains limited. The mechanism underlying heart failure in human patients is related to the altered fat tissue amount, inflammatory processes, and changed cardiac physiology that is additionally complicated by comorbidities [38]. Studies by Hill and Singal [39] on the experimental rat model confirmed that cardiac failure and myocardial infarction in rats are related to the deficits in antioxidant capacity and increased oxidative stress.

The potential antioxidant effects of ASA have yet to be fully understood and confirmed. Pratap et al. [40], in a rat model of cerebral ischemia induced by middle cerebral artery occlusion (MCAO), demonstrated that irbesartan (IRB) and ASA combined, used as pretreatment in MCAO rats, elevated the levels of studied antioxidants, glutathione (GSH), superoxide dismutase (SOD) and catalase. In addition, they showed a significant reduction in thiobarbituric acid reactive substances (TBARS), a marker of lipid peroxidation. The results of their study suggest the antioxidant effects of both IRB and ASA and their synergistic effects. In addition, they described a reduction in brain infarct area volume in animals treated with a combination of IRB and ASA, which may suggest a protective effect of ASA on tissues undergoing ischemia [40]. Berg et al. [41], studying oxidative stress and myocardial injury during coronary artery bypass grafting (CABG), suggested that ASA may exhibit antioxidant effects by enhancing the beneficial effects of the NO axis, inhibiting oxidative stress. This is due to NO’s potential as a cyclooxygenase inhibitor, plasma and tissue oxidases inhibitor, and endothelial nitric oxide synthase activator. In this study, levels of 8-iso-PGF2a, which is an end product of arachidonic acid lipid peroxidation and commonly used as a marker of oxidative stress in serum and urine in cardiovascular diseases, were elevated in patients with the preoperative withdrawal of ASA [41]. The potential antioxidant effect of ASA was also suggested by Zhu et al. [42]. The authors demonstrated that both a combination of ASA and clopidogrel and a mixture of ASA with clopidogrel and Xuesaitong (XST) showed antioxidant effects in the course of cerebral ischemia-reperfusion injury. An inhibitory effect was measured with the concentration of the oxidative stress markers 4-HNE (a marker of lipid peroxidation), protein carbonyl (a marker of protein oxidation), and 8-OHdG (a marker of DNA oxidation) [42]. We observed that non-enzymatic (TAC, TOS, MDA) and enzymatic (GPx, GR, GST) oxidative stress markers were significantly higher in the control group than in the ASA group. Intracoronary ASA administration reduced OS, as measured by selected parameters.

The total antioxidant capacity (TAC) assay is designed to measure different elements of the antioxidant defense system and their ability to neutralize oxidative stress [43]. In this study, the TAC level measured in heart muscle was significantly lower in the infarcted tissue than in the non-infarcted tissue and lower in the control group compared to the ASA treated animals. Meanwhile, TAC levels measured in serum depended on the time of blood sampling during the experiment but not on the type of drug used (ASA vs. NaCl). This shows that ASA had protective effects on the cardiac muscle, significantly improving the different elements of the heart’s antioxidant defense system altogether, while results obtained for serum reflect TAC of the whole body. Interestingly, TAC levels in serum were the highest in the ASA group during reperfusion. Spark et al. [44] examined the TAC of patients with chronic critical leg ischemia undergoing femorodistal bypass. They reported that TAC level in the plasma were reduced in chronic critical ischemia, and the patients presented other evidence of free radical damage, as measured by lipid peroxidation and increased vascular permeability. It is known that ASA pretreatment applied before ischemia inhibits the decrease in ATP (adenosine triphosphate) and pH during ischemia [9]. Pretreatment with ASA had a protective effect against myocardial ischemia lasting 30 min, and ASA tended to maintain the myocardial ATP content through ischemia and reperfusion. Moreover, ASA pretreatment also improved acidosis during ischemia. The possible mechanisms of the ASA protective effect are membrane stabilization, inhibition of prostaglandin production, inhibition of lactic acid production, heart rhythm stabilization, and collateral blood flow promotion [11]. However, the molecular basis and cellular mechanisms of ASA’s cardioprotective effects remain unknown.

Several reports describing antioxidant protection mechanisms against free radical-induced injury include measuring total antioxidant status (TAS) in body fluids. Surekha et al. [45] reported low TAS levels, compared to controls, in patients with myocardial infarction. Fazendas et al. also showed that low TAS plasma levels in MI patients constituted a risk factor for coronary heart disease [46]. Nojiri et al. also demonstrated significantly lower TAS levels, compared to controls, in 31 patients suffering from coronary artery disease (CAD) [47]. On the other hand, Berg et al. reported elevated TAS levels in two groups of patients, those subjected to percutaneous coronary interventions and coronary angiography [48].

It is challenging to assess antioxidant markers separately due to the various antioxidants present in plasma, serum, urine, or other biological fluids. Thus, TAC and TOS measurements have been used to assess the total status of antioxidant and oxidant systems in organisms [25]. In addition, the oxidative stress index (OSI), the total plasma TOS to TAC ratio, have been used to describe the redox status of oxidation and antioxidation processes [49]. In the present study, OSI and TOS levels were significantly higher in animals from the control group, compared to the ASA group. Similar results were reported by Karabacak et al. [50], who showed that OSI and TAS levels were significantly higher in patients with non-ischemic heart failure compared to control subjects. Also, an elevated OSI level was noted in patients with idiopathic dilated cardiomyopathy [51]. Hill and Singal demonstrated that heart failure subsequent to myocardial infarction was associated with an antioxidant capacity deficit and increased oxidative stress [39]. In addition, CAD patients presented significantly higher OSI and TOS and lower TAC levels than the healthy controls. The authors concluded that oxidative stress is an important element of the early onset CAD pathogenesis, particularly among young smokers [52]. In our study, we observed that TAC was reduced in infarcted heart muscle tissue compared to healthy tissue. However, intracoronary ASA administration significantly increased TAC in heart muscle and serum. We showed that ASA helped to reduce TOS and OSI levels after the infarction-reperfusion event, which confirms its beneficial effect on the oxidative status of heart muscle and serum in the porcine ischemia-reperfusion model.

Lipofuscin is an undegradable material composed of oxidized protein and lipid residues. Its accumulation is proportional to mitochondrial changes as organisms age and the intensity of ROS formation [53]. The present study showed that LF was significantly lower in the ASA group than in the control group, and its lowest level was noted during reperfusion.

Malondialdehyde (MDA) is an important biomarker of oxidative stress, especially lipid peroxidation [54]. It is one of the end products of cell membrane lipid peroxidation, and its content directly reflects the extent of lipid peroxidation and, indirectly, cell damage. Acute myocardial infarction (AMI), accompanied by different levels of ischemic myocardial injury and necrotic areas, attracts many neutrophils that release ROS. Cardiomyocytes are very ROS-sensitive, as the heart is an energy-consuming organ. Acute myocardial infarction inevitably causes ischemic–anoxic myocardial injury, which induces intensive ROS production [55]. Since MDA levels correlate with AMI severity, this indicator has been used as a determinant of severe coronary diseases [56]. Moreover, it was also shown that lipid peroxidation levels measured in patients’ plasma positively correlated with the severity of dilated cardiomyopathy symptoms [57,58]. MDA also reflects coronary artery disease severity and plaque sensitivity [59,60]. Moreover, MDA levels are significantly higher in patients with acute coronary syndromes than in healthy controls and are negatively correlated with these patients’ antioxidant status [61]. Finally, Nand et al. [62] reported that MDA levels tripled in AMI patients compared to a control group. Based on MDA levels in the heart tissue, we conclude that intracoronary ASA administration was beneficial for animals subjected to the ischemia-reperfusion procedure.

Myocardium subjected to a ischemia-reperfusion event undergoes complex injury, including injury of the endothelial cells, vascular smooth muscles, conducting tissue, and cardiomyocytes. The endocardium is most prone to these injuries due to local differences in metabolism and energy requirements. Therefore, myocardial injury and tissue necrosis usually originate and expand towards the epicardium. In the myocardium, the oxygen reduction to water happens two ways: primarily via tetravalent reduction of oxygen by the mitochondrial cytochrome oxidase—the pathway generating no intermediates, and in 5% via a univalent reduction that generates ROS such as free radicals: superoxide anions (O_2_^•−^), hydrogen peroxide (H_2_O_2_), and hydroxyl radical (OH^•^) and singlet oxygen ((1)O_2_^•−^) [63]. In physiological conditions, the antioxidant enzymes are primarily found only in the mitochondria or cytosol. During an ischemic episode, the antioxidant enzymes lose their function and leak into the extracellular fluid, and then the enzymes are washed out during the reperfusion. The washout further depletes the ability to control free radical production. Eventually, the uncontrolled production of free radicals on reperfusion (‘respiratory burst’) exceeds the activity of antioxidant enzymes, so ROS production is no longer controlled [63]. Changes in GPX, GR, and GST antioxidant enzymes systems may help protect various tissues and cells from oxidative stress. Up-regulation of either of these enzyme systems may protect against oxidative damage during hearts ischemia-reperfusion episodes. We observed an increase in GPX and GR activity in the infarcted tissue compared to non-infarcted heart tissue. That phenomenon may result from the repeated exposure to the mildly elevated ROS levels produced due to augmented demands for ATP under increased workload conditions of the heart. The increased antioxidant production may result in myocardium adaptation and, consequently, mitigate the damage caused by ischemia-reperfusion injury. However, prolonged exposure to oxidative stress was found to deplete the defense systems, including a decrease in GPx activity [64].

The main limitation of the present study is the use only of colorimetric assays to measure oxidative stress effects. By their nature, these assays are subject to confounding by colored biological fluids. Immunohistochemical techniques, such as Western Blotting (e.g., MDA- or 4HNE-positive proteins) or MS-based evaluation (e.g., 8-isoprostanes) could be considered better methods for assessing the effects of oxidative stress in tissues. However, the more cost-effective and less time-consuming colorimetric methods allow a lot of data to be gathered from a few animal samples.

## 5. Conclusions

The present study demonstrated that increased in vivo lipid peroxidation, measured with malondialdehyde (MDA) and lipofuscin (LF), decreased antioxidants, total antioxidant capacity (TAC) and total oxidative status (TOS), and increased enzyme activity, glutathione reductase (GR), glutathione peroxidase (GPx), and glutathione S-transferase (GST) in infarcted heart tissue and serum are related to myocardial ischemia-reperfusion injury. Intracoronary-administered acetylsalicylic acid (ASA) alleviated the oxidative stress increase, expressed by increased oxidative stress markers and decreased oxidative stress index (OSI). The cardioprotective effect of intracoronary-administered ASA proved in the porcine model gives foundations for developing new therapies for treating ischemia-reperfusion complications in humans.

## Figures and Tables

**Table 1:** Levels of biochemical and morphological blood tests used to assess the health status of pigs (*n* = 13) subjected to a myocardial ischemia-reperfusion protocol assessing the effectiveness of intracoronary administered acetylsalicylic acid (ASA). The results are presented as mean ± standard deviation and were analyzed using two-way ANOVA.

Assay	Group	Before Ischemia	During Reperfusion	Before Euthanasia	Reference Range (Min-Max)
AST [U/L]	Control (*n* = 6)	33.1 ± 9.0	40.5 ± 10.0	35.6 ± 9.6	16–65
	ASA (*n* = 7)	36.9 ± 8.9	42.3 ± 11.7	39.4 ± 22.7	
Urea [mmol/L]	Control (*n* = 6)	5.2 ± 1.0	6.0 ± 1.6	4.8 ± 1.7	3.3–6.6
	ASA (*n* = 7)	5.7 ± 1.0	6.4 ± 0.8	5.2 ± 1.0	
Creatinine [µmol/L]	Control (n = 6)	143.2 ± 40.7	165.5 ± 59.0	115.0 ± 38.3	88–239
	ASA (*n* = 7)	130.7 ± 27.5	155.6 ± 31.0	107.9 ± 35.3	
Fibrinogen [g/L]	Control (*n* = 6)	3.5 ± 0.3	2.8 ± 0.2	4.1 ± 1.2	2–4
	ASA (*n* = 7)	3.7 ± 0.6	2.9 ± 0.6	3.8 ± 0.4	
WBC [10^9^/L]	Control (*n* = 6)	13.8 ± 2.9	–	14.4 ± 6.8	10–20
	ASA (*n* = 7)	14.7 ± 6.8	–	14.5 ± 2.1	
RBC [10^12^/L]	Control (*n* = 6)	6.0 ± 0.2	–	6.0 ± 0.5	5–8
	ASA (*n* = 7)	6.0 ± 0.5	–	6.0 ± 0.7	

Abbreviations: ASA—acetylsalicylic acid, AST—aspartate aminotransferase, RBC—red blood cells, WBC—white blood cells.

**Table 2:** Oxidative stress markers levels in infarcted and the non-infarcted tissues collected from the left ventricle of the heart of pigs (*n* = 13) subjected to a myocardial ischemia-reperfusion protocol assessing the effectiveness of intracoronary administered acetylsalicylic acid (ASA). The results are presented as mean ± standard deviation or median (lower, upper quartile) and were analyzed using two-way ANOVA.

Oxidative Stress Marker	Health Status of the Left Ventricle Tissue	ASA Group (*n* = 7)	Control Group (*n* = 6)	p_tissue_	p_drug_	p_interaction_
TAC [mmol/L]	infarcted	6.4 ± 0.5	4.8 ± 0.9	<0.001	<0.001	<0.001
	non-infarcted	36.2 ± 1.9	29.4 ± 2.2			
TOS [μmol/L]	infarcted	6.1 ± 0.6	12.2 ± 1.4	<0.001	<0.001	<0.001
	non-infarcted	4.6 ± 1.2	6.4 ±0.9			
OSI * [index]	infarcted	93.7 (90.0–103.2)	236.7 (220.7–331.6)	<0.001	<0.001	<0.05
	non-infarcted	11.8 (10.1–14.8)	21.7 (19.6–24.5)			
MDA [μmol/L]	infarcted	3.7 ± 0.3	6.4 ± 0.7	<0.001	<0.001	<0.001
	non-infarcted	0.8 ± 0.2	1.2 ± 0.2			
GPx [IU/mg protein]	infarcted	77.9 ± 3.6	96.5 ± 1.7	<0.001	<0.001	<0.001
	non-infarcted	34.1 ± 2.7	40.1 ± 3.0			
GR [IU/mg protein]	infarcted	3.4 ± 0.3	5.6 ± 0.3	<0.001	<0.001	<0.001
	non-infarcted	2.8 ± 0.5	3.3 ± 0.7			
GST [IU/mg protein]	infarcted	18.4 ± 1.5	18.9 ± 1.0	<0.001	0.929	0.232
	non-infarcted	15.1 ± 0.4	14.7 ± 0.4			

* Log-transformed data; Abbreviations: ASA—acetylsalicylic acid, GPx—glutathione peroxidase, GR—glutathione reductase, GST—glutathione S-transferase, MDA—malondialdehyde, OSI—oxidative stress index, TAC—total antioxidant capacity, TOS—total oxidative status.

**Table 3:** Contrast analysis of oxidative stress markers in infarcted and the non-infarcted tissues collected from the left ventricle of the heart of pigs (*n* = 13) subjected to a myocardial ischemia-reperfusion protocol assessing the effectiveness of intracoronary administered acetylsalicylic acid (ASA).

Oxidative Stress Markers	Health Status of the Left Ventricle Tissue	p_ASA vs. control_	p_infarcted vs. non-infarcted_ in ASA Group	p_infarcted vs. non-infarcted_ in Control Group
TAC [mmol/L]	infarcted	0.770	<0.001	<0.001
	non-infarcted	<0.001		
TOS [μmol/L]	infarcted	<0.01	<0.05	<0.001
	non-infarcted	<0.001		
OSI * [index]	infarcted	<0.001	<0.001	<0.001
	non-infarcted	<0.001		
MDA [μmol/L]	infarcted	<0.001	<0.001	<0.001
	non-infarcted	0.092		
GPx [IU/mg protein]	infarcted	<0.001	<0.001	<0.001
	non-infarcted	<0.01		
GR [IU/mg protein]	infarcted	<0.001	<0.05	<0.001
	non-infarcted	0.075		
GST [IU/mg protein]	infarcted	0.360	<0.001	<0.001
	non-infarcted	0.429		

* Log-transformed data; Abbreviations: ASA—acetylsalicylic acid, GPx—glutathione peroxidase, GR—glutathione reductase, GST—glutathione S-transferase, MDA—malondialdehyde, OSI—oxidative stress index, TAC—total antioxidant capacity, TOS—total oxidative status.

**Table 4:** Oxidative stress marker levels in the serum of pigs subjected to a myocardial ischemia-reperfusion protocol assessing the effectiveness of intracoronary administered acetylsalicylic acid (ASA). The results are presented as mean ± standard deviation and were analyzed using two-way ANOVA.

Oxidative Stress Markers	Group	Before Ischemia	During Reperfusion	Before Euthanasia	p_drug_	p_time_	P_interaction_
TAC [mmol/L]	ASA (*n* = 7)	100.9 ± 1.8	261.1 ± 19.1	221.0 ± 14.9	0.079	<0.001	<0.001
	Control (*n* = 6)	107.0 ± 4.5	240.9 ± 6.3	258.6 ± 20.5			
TOS [μmol/L]	ASA (*n* = 7)	41.2 ± 1.8	34.8 ± 1.7	73.5 ± 3.1	<0.01	<0.001	<0.001
	Control (*n* = 6)	50.5 ± 2.6	40.8 ± 1.0	91.1 ± 2.5			
OSI [index]	ASA (*n* = 7)	40.9 ± 2.1	13.4 ± 1.0	33.4 ± 3.1	<0.001	<0.001	0.072
	Control (*n* = 6)	47.3 ± 2.8	16.9 ± 0.5	35.4 ± 2.7			
LF [RF]	ASA (*n* = 7)	19.3 ± 1.9	10.9 ± 0.8	19.1 ± 2.8	<0.001	<0.001	<0.001
	Control (*n* = 6)	19.0 ±1.4	12.2 ± 0.6	12.5 ± 2.0			

Abbreviations: ASA—acetylsalicylic acid, LF—lipofuscin, OSI—oxidative stress index, RF—relative lipid extract fluorescence, TAC—total antioxidant capacity, TOS—total oxidative status.

**Table 5:** Contrast analysis for repeated measures of oxidative stress marker levels in the serum of pigs subjected to a myocardial ischemia-reperfusion protocol assessing the effectiveness of intracoronary administered acetylsalicylic acid (ASA).

Oxidative Stress Markers	p_ASA vs. control_ before Ischemia	p_ASA vs. control_ during Reperfusion	p_ASA vs. control_ before Euthanasia
TAC [mmol/L]	<0.01	<0.001	<0.01
TOS [μmol/L]	<0.001	<0.001	<0.001
OSI [index]	<0.001	<0.001	0.251
LF [RF]	0.766	<0.01	<0.001

Abbreviations: ASA—acetylsalicylic acid, LF—lipofuscin, OSI—oxidative stress index, RF—relative lipid extract fluorescence, TAC—total antioxidant capacity, TOS—total oxidative status.

## Data Availability

The data it is not publicly available because supporting data cannot be made available openly due to the owning property nature.
